# Case Report: Complicated Meckel Diverticulum Spectrum in Children

**DOI:** 10.3389/fsurg.2021.674382

**Published:** 2021-05-25

**Authors:** Gunadi, Wahyu Damayanti, Robin Perdana Saputra, Ramadhita, Ibnu Sina Ibrohim, Andi Lestiono, Devy Melati, Winda Intan Permatahati, Titis Widowati, Akhmad Makhmudi

**Affiliations:** ^1^Pediatric Surgery Division, Department of Surgery, Faculty of Medicine, Public Health and Nursing, Universitas Gadjah Mada/Dr. Sardjito Hospital, Yogyakarta, Indonesia; ^2^Department of Child Health, Faculty of Medicine, Public Health and Nursing, Universitas Gadjah Mada/Dr. Sardjito Hospital, Yogyakarta, Indonesia

**Keywords:** Meckel diverticulum (diagnosis), Meckel diverticulum (complications), Meckel diverticulum—surgery, children, diagnostic challenges, exploratory laparotomy, segmental small bowel resection and primary anastomosis

## Abstract

**Background:** Meckel diverticulum (MD) is the most common congenital anomaly of the intestines, with an incidence of 2% of the general population. It can present as various clinical features with complications and be life threatening if diagnosis is delayed and treatment late.

**Case Presentation:** We report three pediatric cases with complicated MD: one female presented with small-bowel obstruction, one male with peritonitis, and one female with severe iron-deficiency anemia, without gross gastrointestinal bleeding nor any ectopic gastric mucosa. All patients underwent exploratory laparotomy, segmental small-bowel resection, and primary anastomosis. They successfully recovered and were uneventfully discharged on the fourth, seventh, and 10th postoperative days, respectively.

**Conclusions:** MD can present with various complication spectrums, including small-bowel obstruction, peritonitis, and severe iron-deficiency anemia, which may cause difficulty in definitive diagnosis, particularly in children. Segmental small-bowel resection and primary anastomosis are effective surgical approaches and show good outcomes for MD patients.

## Introduction

Meckel diverticulum (MD) is the most common congenital anomaly of the intestines, with an incidence of 2% of the general population (1, 2). Several studies showed that its frequency is higher in male patients than in females with a ratio of 1.5:1–4:1 (1–5).

Individuals with MD are usually asymptomatic, but they might present with a variety of clinical features, such as small-bowel obstruction, peritonitis, or gross/occult gastrointestinal bleeding/bloody stool (2, 6). Due to its various clinical presentations, it can be misdiagnosed, particularly in pediatric patients. Only 11% of patients with MD are correctly preoperatively diagnosed as MD, while about 11% of MD are misdiagnosed as acute appendicitis (7). The mortality rate of MD is ~6% and often associated with delayed diagnosis and treatment (8). Moreover, the mortality rate of symptomatic MD is 5%, while mortality in elective surgery of asymptomatic MD is 0% (9). Therefore, it is necessary for pediatric surgeons to recognize its various clinical manifestations early (6, 10) and to perform surgical interventions accordingly to avoid morbidity and mortality.

Here, we report three MD cases in children with various clinical presentations and complications: one female presented with small-bowel obstruction, one male with peritonitis, and one female with severe iron-deficiency anemia, without gastrointestinal bleeding nor any ectopic gastric mucosa.

## Case Presentation

### Case 1

**Figure 1:**
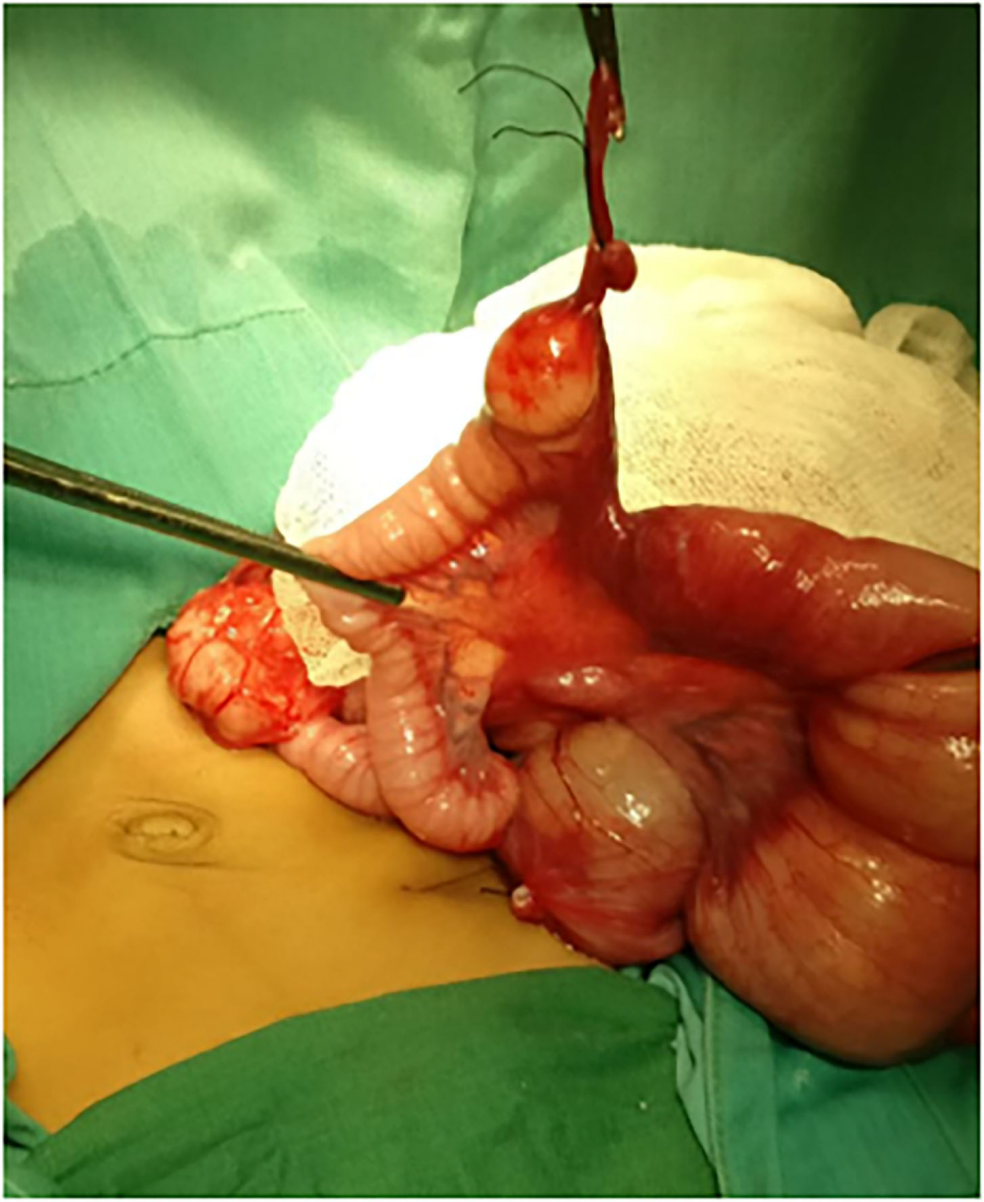
Intraoperative findings revealed a volvulus of fibrous tissue of the vitelline duct and Meckel diverticulum was found.

A 2-month-old female patient was referred to our hospital with complaints of bilious vomiting and abdominal distention. Plain abdominal X-ray showed small-bowel obstruction. Due to the worsening of the clinical manifestations, the patient then underwent an emergency exploratory laparotomy without any further imaging examination. A volvulus of fibrous tissue of the vitelline duct and MD were found (Figure 1). Subsequently, a segmental small-bowel resection with primary anastomosis was performed. The patient recuperated gradually, and on postoperative day (POD) 4, the patient was discharged from the hospital uneventfully.

### Case 2

**Figure 2:**
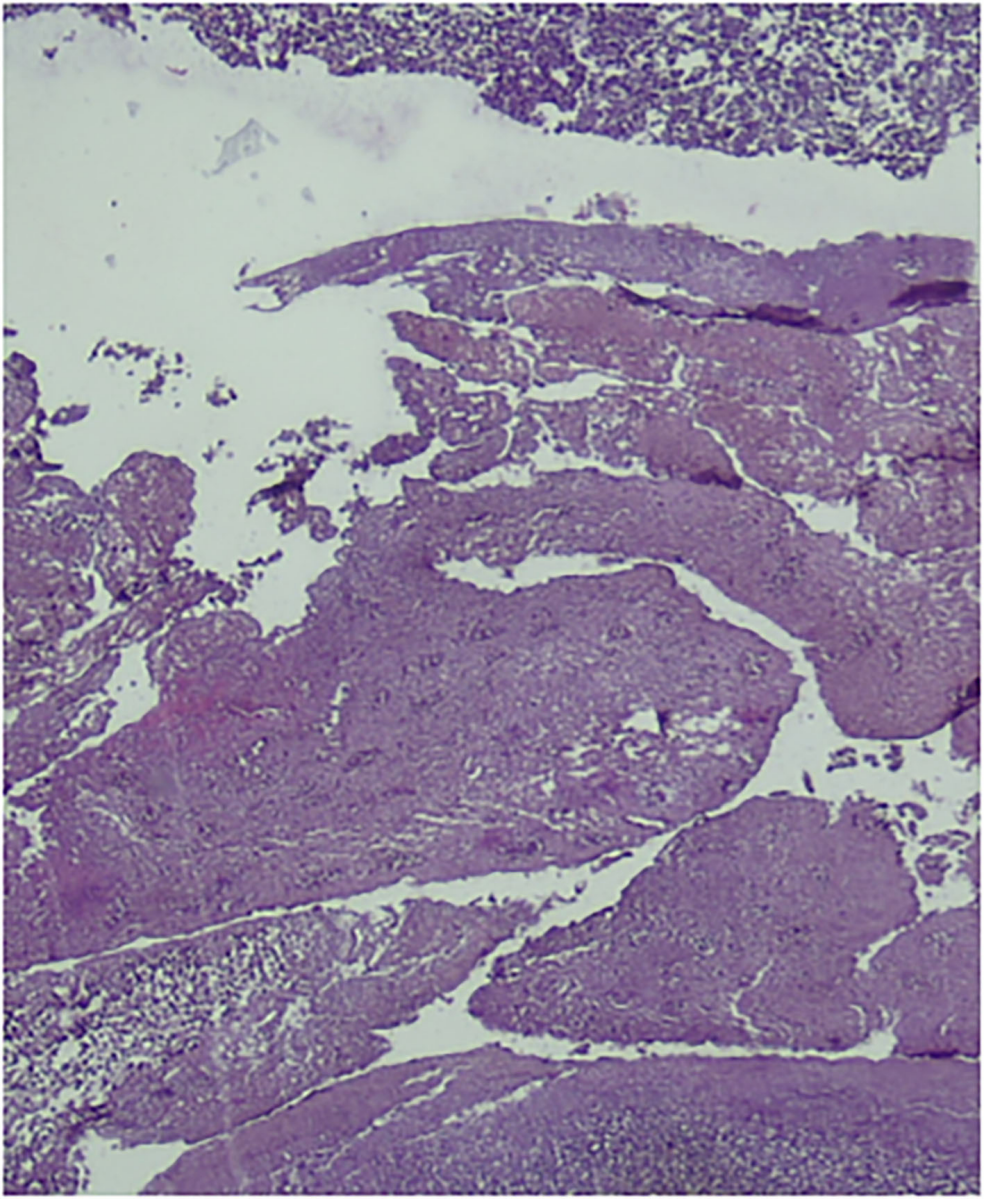
Histopathological findings established a diagnosis of Meckel diverticulum.

A 5-year-old male patient was brought to our hospital with complaints of abdominal pain and fever. Physical examination showed a sign of peritonitis, while abdominal X-rays revealed a small-bowel obstruction. We initially diagnosed him with perforated appendicitis and performed an exploratory laparotomy. During the procedure, we found a perforated MD and an inflamed appendix, but it was not perforated. Next, we conducted segmental small-bowel resection, primary anastomosis, and appendectomy. Histopathological findings established MD (Figure 2). The patient recovered gradually and was discharged from the hospital at POD7 uneventfully.

### Case 3

**Figure 3:**
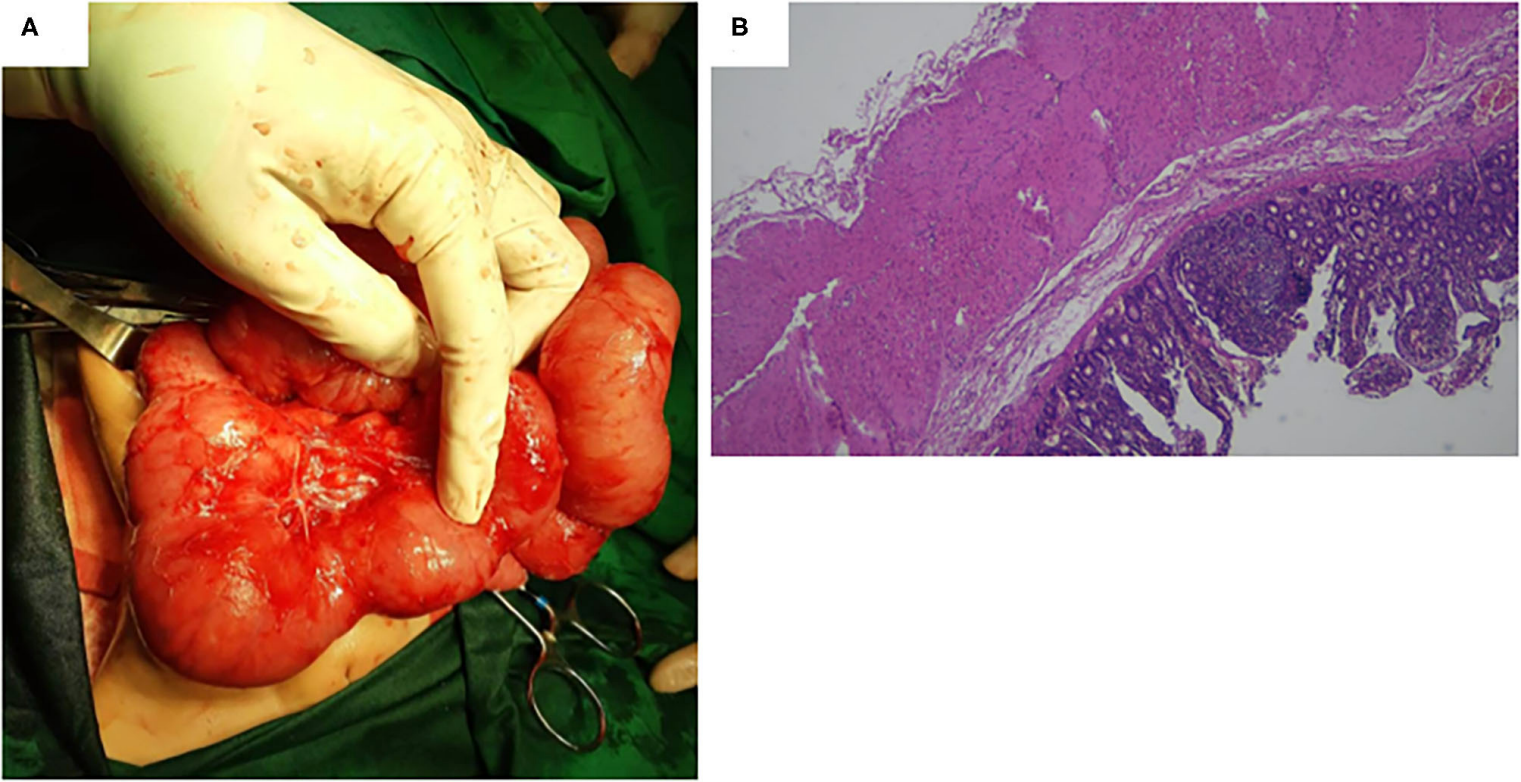
**(A)** A large Meckel diverticulum with a diameter of ~7 cm was found during the surgery. **(B)** Histopathological findings confirmed a Meckel diverticulum, and no ectopic gastric or pancreatic mucosa was found.

A 17-month-old female was referred to us because of severe iron-deficiency anemia. Previously, she had type 3A ileal atresia and underwent ileostomy and stoma closure at 2 days old and 6 months old, respectively, with good outcomes. Since the age of 7 months, she started having severe iron-deficiency anemia without gross gastrointestinal bleeding. Her hemoglobin level decreased continuously by ~2–6 g/dL. She routinely received a blood transfusion due to her severe anemia. She presented a positive benzidine test result for occult blood in her feces. She had been checked for etiologies of severe anemia and showed no hemolytic anemia. We performed an upper gastrointestinal (GI) series and found no abnormality in the upper GI tract. We then decided to perform an exploratory laparotomy and found a large MD with a diameter of ~7 cm (Figure 3A). Segmental small-bowel resection with primary anastomosis was conducted accordingly. Histopathological findings confirmed that MD and no ectopic gastric or pancreatic mucosa were found (Figure 3B). The patient recovered gradually and was uneventfully discharged from the hospital at POD10. However, anemia persisted with the hemoglobin level of 8.2 g/dL at the last follow-up (~15 months after surgery).

## Discussion

In this study, we reported three MD cases in children with various complicated spectra. Patients with MD seldom show a clinical feature; however, they might suffer from various symptoms, including small-bowel obstruction, peritonitis, or severe anemia due to gross/occult gastrointestinal bleeding (6). Therefore, MD should be considered as one of the differential diagnoses of acute abdominal complaints in children (6).

Patients with MD can show symptoms at all ages, with most of them presenting in children, especially <5 years of age (1, 4, 5). Importantly, our patients had a variety of symptoms at various ages: 2 months, 17 months, and 5 years old.

Our first patient revealed a small-bowel obstruction. It was caused by the volvulus of the small-bowel around the diverticular axis. The frequency of intestinal obstruction due to a fibrous band of the vitelline duct as in our first case is very rare (11). However, if the diagnosis and surgical treatment are delayed, the intestines might become gangrenous and the intestinal resection is unavoidable (11).

Our third case suffered from severe iron deficiency anemia. Gastrointestinal bleeding in MD has been proposed because of the damaged intestinal lumen due to the acid produced by ectopic gastric mucosa in MD (1). In this case, no gross intestinal bleeding was found, except occult bleeding shown by the positive benzidine test in her feces, and no ectopic gastric mucosa was present. One of the hypotheses of occult bleeding in MD without ectopic gastric mucosa is inverted MD (12), but this was not the case for our patient. Moreover, the presence of MD might be overlooked during the surgical treatment of intestinal atresia.

There are several etiologies of perforated MD (13), such as irritation from a foreign body, pressure necrosis of the MD wall, or spontaneous perforation due to progressive inflammation of the MD wall, as in our second case. We performed appendectomy in this case as well because of the inflamed appendix. A previous report also performed an appendectomy for a mildly inflamed appendix during the surgical treatment of MD (14).

Since the clinical presentation of MD varies among children, its diagnosis might be difficult, particularly in pediatric populations (8). Without early surgical intervention, the symptomatic MD will lead to complications, including intestinal obstruction, bleeding, and perforation (6). For our third case, we failed to definitively diagnose MD using the upper GI series. This might be due to the barrier of the diverticulum entry by edema (8). Some supporting examinations have been proposed to establish and confirm the diagnosis of MD, including double balloon enteroscopy and video capsule endoscopy (15). Unfortunately, our institution does not have any advanced diagnostic tools for MD, including double balloon enteroscopy and video capsule endoscopy. Accordingly, we decided to perform an exploratory laparotomy for this case. In addition, the persistence of anemia after resection of MD might be the consequence of previous ileal atresia (16) or intestinal malabsorption (17).

There are several surgical approaches for MD, such as segmental small-bowel resection including MD and diverticulectomy only (18). Some surgeons prefer to perform segmental small-bowel resection, including MD, because they want to include any possible intestinal ulceration within the resection samples, while other surgeons choose the latter because they consider that the ulcerated ileum is close to the ectopic gastric mucosa (18). All our patients underwent segmental small-bowel resection with primary anastomosis and showed an uneventful recovery.

In conclusion, MD can present as various complication spectrums, including small-bowel obstruction, peritonitis, and severe iron-deficiency anemia, which may cause difficulty in definitive diagnosis, particularly in children. Segmental small-bowel resection with primary anastomosis are effective surgical approaches and show good outcomes for patients with MD.

## Data Availability Statement

The original contributions presented in the study are included in the article/supplementary material, further inquiries can be directed to the corresponding author/s.

## Ethics Statement

The Medical and Health Research Ethics Committee of Faculty of Medicine, Public Health and Nursing, Universitas Gadjah Mada/Dr. Sardjito Hospital ruled the study exempt from approval because this study was a case series. Written informed consent was obtained from all parents of the patients for publication of this case series.

## Author Contributions

G conceived the study. G and DM drafted the manuscript. G, RS, R, II, AL, and WP collected the data. WD, TW, and AM reviewed the manuscript for important intellectual content. G, WD, TW, and AM facilitated all project-related tasks. All authors agreed to be accountable for all aspects of the work in ensuring the questions related to the accuracy or integrity of any part of the work are appropriately investigated and resolved. All authors read and approved the final manuscript.

## Conflict of Interest

The authors declare that the research was conducted in the absence of any commercial or financial relationships that could be construed as a potential conflict of interest.
